# Genotype–phenotype characterisation of long survivors with motor neuron disease in Scotland

**DOI:** 10.1007/s00415-022-11505-0

**Published:** 2022-12-14

**Authors:** Danielle J. Leighton, Morad Ansari, Judith Newton, David Parry, Elaine Cleary, Shuna Colville, Laura Stephenson, Juan Larraz, Micheala Johnson, Emily Beswick, Michael Wong, Jenna Gregory, Javier Carod Artal, Richard Davenport, Callum Duncan, Ian Morrison, Colin Smith, Robert Swingler, Ian J. Deary, Mary Porteous, Timothy J. Aitman, Siddharthan Chandran, George H. Gorrie, Suvankar Pal, the Lothian Birth Cohorts Group, Sarah Harris, James Prendergast, Tom Russ, Adele Taylor, Ian Deary, and the CARE-MND Consortium, Andrew Bethell, Suzanne Byrne, Gillian Craig, Moira Flett, Hanne Haagendrud, Katarzyna Hafezi, Janice Hatrick, Aidan Hutchison, Helen Lennox, Laura Marshall, Dympna McAleer, Alison McEleney, Kitty Millar, Louise Murrie, David Perry, Gowri Saravanan, Martin Starrs, Susan Stewart, Dorothy Storey, Gill Stott, David Thompson, Carol Thornton, Tanya Van Der Westhuizen, Carolyn Webber

**Affiliations:** 1 School of Psychology & Neuroscience, University of Glasgow, Glasgow, UK; 2 Euan MacDonald Centre for Motor Neuron Disease Research, University of Edinburgh, Edinburgh, UK; 3 Anne Rowling Regenerative Neurology Clinic, Royal Infirmary, Edinburgh, UK; 4 Centre for Clinical Brain Sciences, University of Edinburgh, Edinburgh, UK; 5 Institute of Neurological Sciences, Queen Elizabeth University Hospital, Glasgow, UK; 6 South East Scotland Genetics Service, Western General Hospital, Edinburgh, UK; 7 Centre for Genomic and Experimental Medicine, Institute of Genetics and Cancer, University of Edinburgh, Edinburgh, UK; 8 Institute of Medical Sciences, University of Aberdeen, Aberdeen, UK; 9 Department of Neurology, NHS Highland, Inverness, UK; 10 Department of Neurology, Aberdeen Royal Infirmary, Aberdeen, UK; 11 Department of Neurology, NHS Tayside, Dundee, UK; 12 Lothian Birth Cohorts Group, Department of Psychology, University of Edinburgh, Edinburgh, UK; 13 UK Dementia Research Institute, University of Edinburgh, Edinburgh, UK

**Keywords:** Motor neuron disease, Amyotrophic lateral sclerosis, Survival, Genetics

## Abstract

**Background:**

We investigated the phenotypes and genotypes of a cohort of ‘long-surviving’ individuals with motor neuron disease (MND) to identify potential targets for prognostication.

**Methods:**

Patients were recruited via the Clinical Audit Research and Evaluation for MND (CARE-MND) platform, which hosts the Scottish MND Register. Long survival was defined as > 8 years from diagnosis. 11 phenotypic variables were analysed. Whole genome sequencing (WGS) was performed and variants within 49 MND-associated genes examined. Each individual was screened for *C9orf72* repeat expansions. Data from ancestry-matched Scottish populations (the Lothian Birth Cohorts) were used as controls.

**Results:**

58 long survivors were identified. Median survival from diagnosis was 15.5 years. Long survivors were significantly younger at onset and diagnosis than incident patients and had a significantly longer diagnostic delay. 42% had the MND subtype of primary lateral sclerosis (PLS). WGS was performed in 46 individuals: 14 (30.4%) had a potentially pathogenic variant. 4 carried the known *SOD1* p.(Ile114Thr) variant. Significant variants in *FIG4, hnRNPA2B1, SETX, SQSTM1, TAF15,* and *VAPB* were detected. 2 individuals had a variant in the *SPAST* gene suggesting phenotypic overlap with hereditary spastic paraplegia (HSP). No long survivors had pathogenic *C9orf72* repeat expansions.

**Conclusions:**

Long survivors are characterised by younger age at onset, increased prevalence of PLS and longer diagnostic delay. Genetic analysis in this cohort has improved our understanding of the phenotypes associated with the *SOD1* variant p.(Ile114Thr). Our findings confirm that pathogenic expansion of *C9orf72* is likely a poor prognostic marker. Genetic screening using targeted MND and/or HSP panels should be considered in those with long survival, or early-onset slowly progressive disease, to improve diagnostic accuracy and aid prognostication.

**Supplementary Information:**

The online version contains supplementary material available at 10.1007/s00415-022-11505-0.

## Introduction

**Fig. 1:**
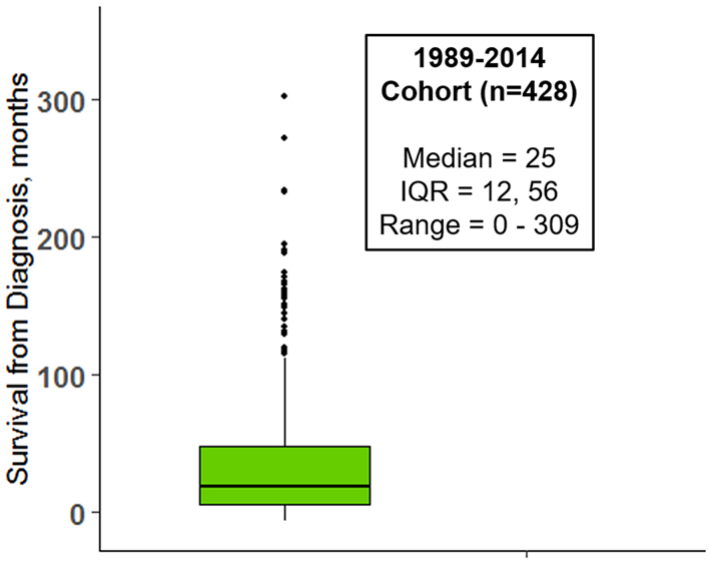
Boxplot of survival from diagnosis (months) in the 1989–2014 genotyped cohort (*n* = 428) [5]

The Scottish Motor Neuron Disease Register (SMNDR) (re-launched as the Clinical Audit Research and Evaluation for MND (CARE-MND) platform in 2015) has been collecting data regarding people living with MND (amyotrophic lateral sclerosis (ALS) and MND subtypes [primary lateral sclerosis (PLS), primary muscular atrophy (PMA) and progressive bulbar palsy (PBP)] in Scotland since 1989 [1, 2]. The unique longevity of the register and the united efforts of the CARE-MND Consortium have provided extensive insight into the phenotypic and genetic heterogeneity of the disease [1, 3, 4]. Recent analysis of a historical cohort of 428 Scottish people with MND indicated a median survival of 3.5 years from onset of symptoms and 2 years from diagnosis [5]. However, the upper range of survival was 25.8 years from diagnosis (Fig. 1).

There is currently no definition of long survival with MND, with literature ranging from 5 to 10 years [6–9]. Previous European estimates of patients surviving more than 10 years from diagnosis are 11.8% [8]. The phenotype is variable: some long-surviving people with MND have typical features of ALS [6, 7], while others have lower frequency of bulbar symptoms [2, 10] or specific disease subtypes such as PLS [11]. Individuals with PLS are thought to have younger onset disease [12]. Prediction of long survival is otherwise challenging; this prognostic uncertainty compounded by a typically protracted time to reach a diagnosis is psychologically difficult for people with MND and their families [12].

Some *SOD1* variants and a specific variant in the *UNC13A* gene (rs10419420) are thought to impart long survival [13, 14]. People with apparent PLS have been found to have genetic variants not typically associated with MND, such as those normally associated with hereditary spastic paraplegia or Parkinson’s disease [11, 12, 15]. Otherwise, ‘long survivors’ have not been extensively genotypically characterised.

Harnessing 30 years of data from the Scottish MND Register/CARE-MND platform, we aimed to study long-survivors phenotypically and genetically. Whilst the overall proportion of long-survivors in MND populations is low, improved phenotypic understanding of this group is important to improve early and accurate diagnosis. Characterisation of long survivors might help to counsel patients and prognosticate at diagnosis. Long survivors also comprise a significant proportion of the prevalent clinical and ‘research-ready’ population and so knowledge of disease features may minimise bias and optimise generalisability of clinical trials [8]. DNA sequencing may provide clues towards genotype–phenotype associations, protective genetic factors or phenotypic overlap with disorders such as hereditary spastic paraplegia (HSP) [16]. We therefore aimed to identity phenotypic markers or genetic variants that might distinguish long survivors from a typical incident population of people with MND in Scotland, to guide clinical management and provide recommendations for genetic testing.

## Methods

### Recruitment and ethical approvals

Patients were recruited via the Scottish MND Register/CARE-MND Platform (ethical approvals MREC/98/0/56 1989–2010, 10/MRE00/78 2011–2015, and the Scotland A Research Ethics Committee 15/SS/0126 2015 onwards). DNA samples were donated to the Scottish MND DNA Bank and the Scottish Regenerative Neurology Tissue Bank (MREC/98/0/56 1989–2010, 10/MRE00/77 2011 to 2013, 13/ES/0126 2013–2015, 15/ES/0094 2015-present). The Lothian Birth Cohorts (LBC) – a research population of Scottish adults born in 1921 and 1936—were used as ancestry-matched genetic controls [17]. Ethical permission for the LBC1936 study protocol was obtained from the Multi-Centre Research Ethics Committee for Scotland (Wave 1: MREC/01/0/56), the Lothian Research Ethics Committee (Wave 1: LREC/2003/2/29), and the Scotland A Research Ethics Committee (Waves 2, 3, 4 and 5: 07/MRE00/58). Ethical permission for the LBC1921 study protocol was obtained from the Lothian Research Ethics Committee (Wave 1: LREC/1998/4/183; Wave 2: LREC/2003/7/23; Wave 3: LREC1702/98/4/183), the Scotland A Research Ethics Committee (Waves 4 and 5: 10/MRE00/87).

### Patient details

Long survivors were defined as people with MND with survival from diagnosis beyond the 80th percentile of the historical 1989–2014 Scottish genotype study cohort (> 8 years) [5]. This cut-off was determined as a conservative measure of long survival based on previous literature [6–8]. Survival was calculated from date of symptom onset/diagnosis until death or censorship date (3rd August 2021). The following phenotypic data fields were available for analysis: Sex, Age of Onset, Age of Diagnosis, Time to Diagnosis, Survival from Onset, Site of Onset, El Escorial Classification, Family History of MND, Feeding Tube Insertion, Non-invasive Ventilation (NIV) Use, Riluzole Use. Rate of change of the ALS-Functional Rating Scale (ALS-FRS) was not thought to be informative as it is considered inadequate for upper motor neuron predominant MND such as PLS [18]. Other predictors, such as cognitive assessments, were only available for a small number of long surviving patients and so could not be included in statistical analyses.

### Genetic analysis

**Fig. 2:**
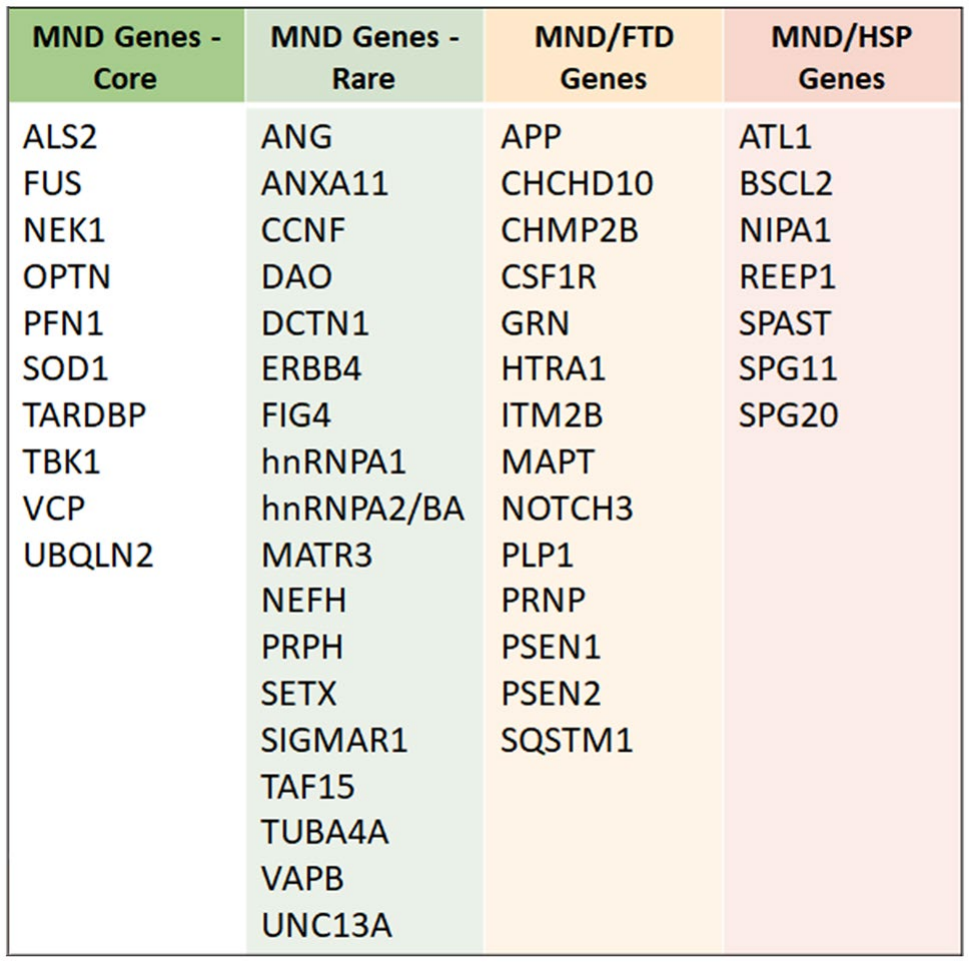
Motor neuron disease (MND)-associated genes. (i) Core MND-associated genes, (ii) Rare MND-associated genes, (iii) Genes associated with MND with frontotemporal dementia (MND-FTD), FTD or FTD mimics, (iv) Genes associated with hereditary spastic paraplegia (HSP) or MND-HSP overlap syndromes

Patient DNA samples were analysed using whole genome sequencing technology, performed as part of the Scottish Genomes Partnership (SGP) study [19]. Samples were sequenced to 30X coverage using TruSeq Nano library preparation kits and a HiSeq X sequencing platform (Illumina). FASTQ files were aligned to the human genome build GRCh37 using bwa mem (0.7.13) [20]. Post-processing was performed with samblaster (0.1.22) [21] to mark duplicate reads, and the Genome Analysis ToolKit (GATK, v3.4-0-g7e26428)[22] for indel realignment and base recalibration. Genotype likelihoods for each sample were calculated using the GATK HaplotypeCaller and resulting GVCF files were called jointly using GATK’s GenotypeGVCFs function. Variant quality score recalibration (VQSR) was performed as per GATK best-practices [23] and a truth sensitivity threshold of 99.9% applied. Variants were filtered to include only those present in 49 MND-associated/MND-mimic genes (Fig. 2). Filtered variants were annotated and population frequency filters were applied using VarSeq Golden Helix software [24] to include only those with minor allele frequency (MAF) ≤ 0.01 in gnomAD 2.0.1v3 [25]. Variants were annotated using multiple in silico prediction algorithms from the Database for Nonsynonymous SNPs and their Functional Predictions (dbNSFP) [26] and included: SIFT, PolyPhen2 HDIV and HVAR, Mutation Taster, Mutation Assessor, FATHMM, PROVEAN, GERP and PhastCons. Measures of impact on splice site included scores derived from adaptive boost (Ada) and random forest (RF) models [27]. Samples were also tested for *C9orf72* hexanucleotide repeat expansions using repeat prime PCR methodology; ≥ 30 repeats was considered pathogenic [28].

Variants were classified using the American College of Medical Genetics and Association for Molecular Pathology (ACMG-AMP) framework and adhering to the Association for Clinical Genomic Science (ACGS) UK 2020 guidelines [29, 30]. Co-segregation was determined using methods described by Jarvik et al. [31] DNA samples from the Lothian Birth Cohorts (*n* = 1385) were used as ancestry-matched controls to identify variants enriched in cases versus controls as per ACMG-AMP guidelines. The gnomAD database was used as a population control data set [25]. Variants were considered significantly more prevalent in cases versus controls if odds ratio > 5.0 and confidence intervals did not cross 1.0 (ACMG-AMP criteria PS4) [29]. Additionally, gnomAD was used as a population control data set (ACMG criteria PM2) [25]. Variants meeting criteria thresholds for a pathogenic or likely pathogenic classification were reported. Variants of uncertain clinical significance (VUS) that fulfilled some criteria for being pathogenic, without reaching strict ACMG-AMP thresholds for significance, were considered under the Bayes rules outlined by Tavtigian et al. to determine a posterior probability of their being potentially pathogenic (probability > 0.5) [32].

### Statistical analyses

Long survivors were compared with the incident population cohort of people with MND in Scotland diagnosed in 2015–17 (*n* = 437) using univariate statistics (Fisher’s exact tests, t-tests and Wilcoxon rank-sum tests). Correction for multiple testing was undertaken using the Bonferroni method. R statistical programming was used [33].

## Results

### Phenotypes

Fifty eight long survivors were identified, representing 3.3% of the total number of individuals on the CARE-MND platform at the time of data acquisition (*n* = 1779). Median survival from onset was 18.3 years (IQR 14.6–22.4) (missing dates for four individuals); from diagnosis 15.5 years (IQR 12.0–19.0). Thirty seven (63.8%) people were alive at censorship date, giving a point prevalence of 8.9% (Scottish prevalence of MND = 415). Fifty-four (90%) had consented to research and were characterised phenotypically. Percentage completed data per variable ranged from 94.4 to 100%. Phenotypic characteristics are outlined in Table 1. Male-to-female ratio was 2:1. Twenty-nine (54.7%) of long survivors had ALS, 22 (41.5%) PLS, one individual presented with Monomelic Amyotrophy (Other) and one with Progressive Bulbar Palsy (PBP). The most common site of onset of disease was lower limbs (55.8%). Six (11.5%) had a family history of MND, although two individuals were related. Of the 13 (25%) on ventilation, 12 of these were non-invasive and one invasive ventilation.

**Table 1:** Comparison of phenotypic characteristics between long survivors and incident cohort of people with motor neuron disease

Phenotypic characteristics	Incident cohort (*n* = 437)	Long survivors (*n* = 54)	Statistical test *p*-value
Sex: Male (%)	275 (62.9)	36 (66.6)	Fisher's *p = 0.66*
Ethnicity: White Scottish/British/Other (%)	415 (98.6)	51 (100)	Fisher's *p = 0.76*
Mean age of onset, years (SD)	63.9 (10.9)	47.4 (13.6)	t-test ***p* < *0.0001***
Mean age of diagnosis, years (SD)	65.5 (10.7)	51.5 (13.5)	t-test ***p* < *0.0001***
Median time to diagnosis, months (IQR)	12.0 (8.0–23.0)	26.0 (13.0–45.0)	Wilcoxon ***p* < *0.0001***
Site of Onset			Fisher's p = 0.038
Bulbar (%)	123 (28.5)	14 (26.9)	
Upper limb (%)	103 (23.8)	8 (15.4)	
Lower limb (%)	144 (33.3)	29 (55.8)	
Mixed (%)	41 (9.5)	1 (1.9)	
Cognition (%)	12 (2.8)	0 (0)	
Respiratory (%)	5 (1.2)	0 (0)	
Other (%)	4 (0.9)	0 (0)	
Classification			Fisher's p < 0.0001
ALS (%)	338 (77.4)	29 (54.7)	
PBP (%)	25 (5.7)	1 (1.9)	
MND-FTD (%)	25 (5.7)	0 (0)	
PLS (%)	19 (4.3)	22 (41.5)	
PMA (%)	19 (4.3)	0 (0)	
Other (%)	11 (2.5)	1 (1.9)	
*Family history of MND: Yes (%)*	39 (9.2)	6 (11.5)	Fisher's *p* = *0.66*
Taking Riluzole: Yes (%)	173 (40.0)	32 (62.7)	Fisher's ***p* = *0.0005***
Feeding Tube Inserted: Yes (%)	136 (31.3)	6 (11.8)	Fisher's ***p* = *0.0003***
Non-invasive/Invasive ventilation commenced: Yes (%)	118 (27.2)	13 (25.0)	Fisher's *p* = *0.15*

*ALS* amyotrophic lateral sclerosis, *PBP* progressive bulbar palsy, *MND-FTD* motor neuron disease with frontotemporal dementia, *PLS* primary lateral sclerosis, *PMA* progressive muscular atrophy

Significant *p*-values in bold

Bonferroni corrected threshold for significance was 0.0045. Long survivors were significantly younger at onset (47.4 years) and diagnosis (51.5 years) compared with incident people with MND (*p* < 0.0001). Time to diagnosis was significantly more prolonged (26 months versus 12 months; *p* < 0.0001). Classification of disease was significantly different (*p* < 0.0001), with long survivors more likely to have PLS than incident patients (41.5% of long survivors). Although long survivors were more likely to have lower limb onset disease, this did not reach statistical significance. Long survivors were more likely to be prescribed riluzole at any point (62.7%; *p* = 0.0005) but were significantly less likely to undergo gastrostomy insertion (only 11.8%; *p* = 0.0003) (Table 1).

### Genotype

Forty six (79.3%) of all long survivors donated a DNA sample. Forty six unique variants (94 variant calls including variants found in multiple individuals) met filtering criteria (Supplementary Table 1). All variants were detected in a heterozygous state. Three were considered likely benign variants (6.5%). Sixteen variants fulfilled ACMG-AMP/ACGS criteria for being likely pathogenic (34.8%). However, three variants were in genes normally causing disease in an autosomal recessive pattern (*ALS2* and *SPG11*) and three variants were novel frameshift/indel variants in a likely unstable region of a tenuously associated MND gene (*TAF15*) (Supplementary Table 1). Excluding these variants, 10 were classified as likely pathogenic (21.7%) (Table 2). The remaining 27 variants (58.7%) were classified as VUS; two of these (4.3% of all variants) leaned towards the pathogenic end of the spectrum with a supportive posterior probability (> 0.5) (Supplementary Table 1) [32]. In contrast, 21 VUS leaned towards the benign end of the spectrum (45.7%). The remaining four variants (8.7%) met both benign and pathogenic criteria, with a posterior probability of 0.5 (Supplementary Table 1). A total of 14 individuals had at least one variant fulfilling criteria for being pathogenic or likely pathogenic variant (30.4% of cohort). A total of 17 patients carried at least one pathogenic or likely pathogenic variant *or* a VUS which fulfilled some pathogenic criteria (posterior probability > 0.5) (37.0% of cohort) (Supplementary Table 1).

**Table 2:** Significant variants identified in long survivors with motor neuron disease (MND)

Gene	Genomic position (GRCh37)	HGVS coding transcript/variant DNA change	Amino acid change	Variant type	Number cases	Number controls	ACMG-AMP/ACGS Evidence
*FIG4*	6:110113822A > T	NM_014845.5:c.2414A > T	p.(Asn805Ile)	Missense	1	1	PS4, PM2
*HNRNPA2B1*	7:26240202G > A	NM_031243.2:c.-5C > T	*5’ UTR*	5’ UTR Variant	1	0	PS4, PM2
*SETX*	9:135211747C > G	NM_015046.5:c.654G > C	p.(Lys218Asn)	Missense	1	3	PS4, PM2
*SOD1*	21:33039672 T > C	NM_000454.4:c.341 T > C	p.(Ile114Thr)	Missense	4*	0	PS4, PM2, PP1 Moderate, PP3
*SOD1*	21:33036142G > A	NM_000454.4:c.112G > A	p.(Gly38Arg)	Missense	1	0	PS4, PM2, PP3
*SPAST*	2:32366960 T > -	NM_014946.3:c.1494-3delT	*Intronic*	Intronic splice region	2	0	PS4, PM2, PP3
*SQSTM1*	5:179260112GAG > -	NM_003900.4:c.835_837delGAG	p.(Glu280del)	Inframe deletion	1	0	PS4, PM2, PP3
*SQSTM1*	5:179263547C > T	NM_003900.4:c.1277C > T	p.(Ala426Val)	Missense	1	2	PS4, PM2
*TAF15*	17:34171667A > G	NM_139215.2:c.1364A > G	p.(Tyr455Cys)	Missense	1	0	PS4, PM2, PP3
*VAPB*	20:56964578G > A	NM_004738.4:c.58 + 5G > A	*Intronic*	Intronic splice region	1	0	PS4, PM2, PP3

Classification of pathogenicity based on use of American College of Medical Genetics and Association for Molecular Pathology (ACMG-AMP) and Association for Clinical Genomic Science (ACGS) UK 2020 guidelines. All variants detected in heterozygous state. Genomic controls comprised ancestry-matched individuals from the Lothian Birth Cohorts (*n* = 1385). Variants were considered significantly more prevalent in cases versus controls if odds ratio > 5.0 and confidence intervals did not cross 1.0 (ACMG-AMP criteria PS4). Additionally, gnomAD was used as a population control data set (ACMG criteria PM2). Co-segregation was quantified using methods described by Jarvik et al. (PP1). In silico predictions were evaluated using VarSeq Golden Helix annotations (PP3)

*UTR* untranslated region

*Indicates that 2 of the cases are related

Pathogenic or likely pathogenic variants included four incidences of the *SOD1* p.(Ile114Thr) Scottish founder mutation (Table 2) [3, 5]. One of the p.(Ile114Thr) variants and the *SOD1* p.(Gly38Arg) variant (Table 2) were observed in the Scottish population previously [5]. Variants of interest were also identified in *FIG4, hnRNPA2B1, SETX, SPAST* and *VAPB* genes (Table 2). Two related individuals had both the *SOD1* p.(Ile114Thr) mutation plus a frameshift mutation (p.Asp449Thrfs*28) in the *TAF15* gene [34]. The *TAF15* mutation was found in two unrelated samples and nine controls, giving an odds ratio of 6.9 (95% CI 1.5–33.1). However, other variants were also observed at the same site as this loss-of-function variant suggesting possible instability of the region (Supplementary Table 1). Mutations in *TAF15* are postulated to be linked with MND pathogenesis due to their shared role in FET (*FUS, EWSR1, TAF15*) protein pathways but are not clearly associated with MND and so the significance of this variant is attenuated [34].

No patients in this cohort had expansions in the *C9orf72* hexanucleotide sequence within the pathogenic range.

### Genotype–phenotype associations

Four individuals had the *SOD1* p.(Ile114Thr) Scottish founder variant [3, 5], (Table 3) although two of these were first cousins once removed and had another family member with MND suggesting moderate co-segregation of this variant as per calculations outlined by Jarvik et al. (Table 4) [31]. All four patients had familial lower limb onset ALS with a median age of onset of 31.5 years and all required NIV but not gastrostomy. Case note review did not reveal significant cognitive impairment. Another individual had the *SOD1* p.(Gly38Arg) variant; this variant is associated with a mouse model of ALS which recapitulates some of the motor features [35]. However, there is a paucity of knowledge regarding its phenotypic correlates. This individual had a family history of MND—his father developed symptoms of MND in his 30s and died in his 60s, implying a similar course of disease.

**Table 3:** Key phenotypic characteristics of long survivors with variants of interest

	*FIG4* p.(Asn805Ile) (*n* = 1)	*HNRNPA2B1* 5 ‘ UTR c.-5C > T (*n* = 1)	*SETX* p.(Lys218Asn) (*n* = 1)	*SOD1* p.(Ile114Thr) (*n* = 2)	*SOD1* p.(Gly38Arg) (*n* = 1)	*SPAST* Intronic c.1494-3delT (*n* = 2)	*SQSTM1* p.(Glu280del) (*n* = 1)	*SQSTM1* p.(Ala426Val) (*n* = 1)	*TAF15* p.(Tyr455Cys) (*n* = 1)	*VAPB* Intronic c.58 + 5G > A (n = 1)
Sex	Male	Male	Female	Male Male	Male	Male Male	Male	Male	Male	Male
Ethnicity	White Scottish	White Scottish	White Scottish	White Scottish White Other	White Scottish	White Scottish White Other	White Scottish	White Scottish	White Scottish	White Scottish
Age of onset, years	54	45	41	31 48	31	76 65	28	53	49	37
Time to diagnosis, months	30	13	78	46 41	5	11 4	83	66	159	17
Survival from onset, months	175	143	355	199 149	245	164 253	223	216	308	211
Site of onset	Lower limb	Lower limb	Bulbar	Lower limb Lower limb	Upper limb	Lower limb Lower limb	Lower limb	Upper limb	Lower limb	Lower limb
MND classification	ALS	PLS	PLS	ALS ALS	ALS	PLS ALS	ALS	ALS	PLS	PLS
Family history of MND	No	No	No	Yes Yes	Yes	No No	No	No	No	No
Taking riluzole	No	No	No	No Yes	Yes	No Yes	Yes	Yes	No	Yes
Feeding tube	No	Yes	No	No No	No	No No	Yes	Yes	No	No
Non-Invasive/Invasive ventilation	Yes	No	No	Yes Yes	No	No No	Yes	Yes	No	No

*ALS* amyotrophic lateral sclerosis, *PLS* primary lateral sclerosis

**Table 4:** Key phenotypic characteristics of two related long-survivors

	CASE 1*: *SOD1* p.(Ile114Thr)	CASE 2*: *SOD1* p.(Ile114Thr)
Sex	Female	Male
Ethnicity	White Scottish	White Scottish
Age of Onset, years	32	23
Time to Diagnosis, months	38	37
Survival from Onset (months)	232	263
Site of Onset	Lower limb	Lower limb
Classification	ALS	ALS
Family History of MND	Yes	Yes
Taking Riluzole	No	No
Feeding Tube	No	No
Non-Invasive/Invasive Ventilation	Yes	Yes

*ALS* amyotrophic lateral sclerosis

Two patients had missense mutations in *FIG4* and *SETX* respectively, the latter in a patient with PLS. These genes have been associated rarely with both ALS and PLS [15]. Two patients had *SQSTM1* variants; these have been described in familial and apparently sporadic MND, as well as in frontotemporal dementia (FTD) without MND, and are also associated with multisystem disease [36]. One individual had a variant in *hnRNPA2B1;* although this variant was in the 5’UTR (untranslated region) of this gene, the region is highly conserved and the variant was absent from controls with a low MAF in population databases. Variants in *hnRNPA2B1* are associated with inclusion body myopathy with early-onset Paget disease with or without frontotemporal dementia, but have been described in ALS [37, 38]. On case note review, neither patient with the *hnRNPA2B1* variant or the *SQSTM1* variant had evidence of multisystem disease typical of the genes (inclusion body myopathy or Paget’s disease). However, the patient with the *hnRNPA2B1* variant had progressive cognitive impairment/dementia but was determined to have a PLS phenotype. The two patients with *SQSTM1* mutations had limb onset ALS, requiring both NIV and gastrostomy. One of these patients was also a heterozygous carrier of a loss of function variant in the *SPG11* gene (p.M245Vfs*2) (Supplementary Table). This *SPG11* variant has previously been reported in ClinVar[39] in a biallelic state associated with HSP and juvenile ALS but has not previously been observed in *trans* with another variant. The patient, however, had generalised signs and symptoms in-keeping with a classical ALS phenotype, with supportive electromyography (EMG) and gastrostomy and NIV requirements. This patient also underwent post-mortem examination and had typical ALS-associated TDP-43 intracytoplasmic aggregates [Dr Jenna Gregory et al., unpublished].

Two patients carried a *SPAST* intronic variant; this variant was absent from controls and gnomAD but has been reported in Clinvar associated with autosomal dominantly inherited HSP (SPG4) and had supportive in silico predictors of an effect on splicing [40]. Interestingly, the two patients harbouring this variant had limb onset ALS and PLS and did not require NIV or gastrostomy suggesting perhaps misdiagnosis of MND or an overlapping phenotypic spectrum. A further splice site mutation was observed in the *VAPB* gene in an individual with PLS. *VAPB* variants are rare in MND and the gene is small and tolerant to change. However, all splice algorithms predicted a significant reduction in splice efficiency and the variant was absent in controls with a low MAF.

## Discussion

The prevalence of long survivors in the Scottish population (8.9%) is comparable with previous European estimates [8]. Patients in this cohort are younger at onset and diagnosis and have a longer time to diagnosis. We suspect that the diagnostic delay reflects uncertainty based on gradual evolution of clinical features and absence of lower motor neuron features on EMG. These findings agree with previously published observations [7–9, 12, 41]. However, this delay presents a window for improvement in MND care.

Our cohort is enriched for people diagnosed with upper motor neuron predominant MND (PLS). Although not statistically significant compared with incident patients, the male-to-female ratio in the long-surviving group was high (2:1) and this may be due to the contribution of PLS cases which are known to be largely male (ratio 2–4:1) [12]. That PLS and ALS are on a continuum of disease has long been debated; however, recent systematic reviews found no consistent distinguishing imaging or pathological biomarkers [12, 18].

Long survivors are more likely to start on riluzole than incident patients. This may be related to opportunity in the context of long duration of disease. While riluzole has been trialled predominantly in people with ALS, an appeal was made to the National Institute for Clinical Excellence (NICE) in 2001 to allow its use in other forms of MND [42]. Clearly, in current practice, patients with PLS do receive riluzole. Long survivors are significantly less likely to have a gastrostomy inserted, in spite of there being more opportunity, temporally, for this to occur. However, the proportions of bulbar-onset patients in incident and long surviving cohorts are comparable (28.5% and 26.9% respectively). It is possible that upper motor neuron pseudobulbar symptoms (for example, dysarthria) in the long-surviving PLS cohort are being confused for true bulbar symptoms. It may be clinically difficult to localise dysarthria clinically, especially early in disease. However, dysphagia is rare in PLS [43]. Indeed, only two (14.3%) patients with bulbar-onset disease had gastrostomy insertion; other patients were noted to have dysarthria with preserved swallow. Although we might expect respiratory muscle weakness to be associated with poor outcome, a surprisingly high proportion of long survivors required NIV (25.0% in the long survivors, 27.2% in the incident cohort). It was unclear if this requirement occurred late in disease or if NIV contributed to survival. Long surviving people with MND form a significant proportion of the prevalent population; exclusion of such participants from MND clinical trials risks discrimination and attenuation of population for recruitment and study power. Our data suggest that detailed characterisation of such participants (by classification and by distinguishing bulbar from pseudobulbar symptoms) would aid trial inclusion, generalisability and interpretation.

Only 11.5% of the long surviving cohort had a family history of MND whereas almost a third (30.4%) of the genotyped cohort had a ACMG-AMP likely pathogenic variant. As would be expected, this is higher than when a limited six-gene panel was employed for an unselected cohort of MND patients in Scotland (17%) [5]. Genetic analysis in long survivors broadened the already wide phenotypic spectrum of disease of patients with the known pathogenic *SOD1* p.(Ile114Thr) variant which is frequently observed in the Scottish population [5]. Families with this variant in Scotland may be encouraged that it can be associated with long survival. As previously described, people with this variant have an otherwise homogeneous phenotype with limb-onset ALS, preserved bulbar function and low gastrostomy uptake and absence of cognitive impairment.

The presence of other rare variants in MND-associated genes (*FIG4, hnRNPA2B1, SETX, SQSTM1, TAF15, VAPB*) in patients with both ALS and PLS without a family history confirms the sporadic nature of variants in these genes in the Scottish population. Although these variants met ACMG-AMP/ACGS classification criteria for being likely pathogenic, it is not possible to determine if they are disease-causing in a rare heterogeneous condition such as MND. We raise the possibility that people with MND carrying mutations in the *SPAST* gene may have an MND syndrome similar to HSP. In general, HSP will normally present earlier than upper motor neuron predominant ALS/PLS and patients are more likely to have lower limb onset disease and a family history; however, there will be exceptions and overlap [12, 16]. Extended and explicit past medical history and family history questioning (including inclusion body myopathy, Paget’s disease, FTD, HSP and other neurological causes of spasticity) might have illuminated patterns in-keeping with the identified genetic variants.

We have also highlighted VUS of potential interest which meet some pathogenic criteria without fulfilling strict ACMG-AMP requirements for pathogenicity; these require future study and reassessment.

The absence of *C9orf72* pathogenic expansions in this study is supportive of this variant typically being a poor prognostic marker [44]. Indeed, no long survivors were diagnosed with MND-FTD, compared with 5.7% of the incident cohort. Formal systematic cognitive testing was not undertaken for the majority of long survivors, however, as this assessment tool was only routinely applied within the last decade.

### Limitations

Our study would benefit from inclusion of cognitive profiling. Details regarding time to intervention (gastrostomy, NIV) were not available for all patients but future analysis of long survivors might study the temporal relationship of these measures on outcome. Our control population is ancestry but not age or sex-matched and so may not reflect the characteristics of our typically young MND long survivors.

We have reported variants as per ACMG-AMP guidelines. MND-associated variants are rare and have variable penetrance and so often struggle to fulfil strict ACMG-AMP criteria. Many likely pathogenic variants were so defined due to their absence in controls and absence in gnomAD alone. However, these guidelines provide a necessary structure for variant assessment and allow us to report variants which might as yet fail to achieve clinical significance but might be of research interest. As this was a research project and results were not relayed to patients or families, frameshift and indel variants were not confirmed by Sanger sequencing and so it is not possible to determine if they are truly disruptive. Our 49-gene panel was comprehensive for MND-spectrum genes but we did not examine the androgen receptor CAG trinucleotide repeat associated with Kennedy disease [7], nor did we exhaust all rare HSP and PLS-associated genes [12]. Pathology correlation was only available for one patient in this cohort. Although CARE-MND recruitment methods are stringent [1], it is possible that some patients are misdiagnosed and pathological examination would provide confirmation of disease in rare gene and MND-mimic gene carriers [18].

## Implications and conclusions

With the benefit of three decades of longitudinal data collection through the Scottish CARE-MND database, we have shown that long surviving people with MND can be characterised by younger age at onset and diagnosis, increased incidence of PLS and, crucially, a longer time to diagnosis (median 2.2 years, upper range 15 years). This long period of diagnostic uncertainty for a typically young person is a key target for improvement in care; such patients may otherwise be denied access to designated benefits and specialised support. Early clinical genotyping of such individuals may help to provide reassurance – for example, the absence of a *C9orf72* pathogenic expansion and presence of the *SOD1* p.(Ile114Thr) mutation or other rare mutation might indicate better outcome. Additionally, presence of the *SOD1* p.(Ile114Thr) variant might imply long preservation of bulbar function and cognitive function but allow individuals to prepare for NIV requirement. Extended family history, including multisystem disease and neurological syndromes featuring UMN signs/spasticity, might be of particular merit in those with young onset, slowly progressive ALS or PLS; in these cases, MND and HSP genetic testing may be appropriate. Long survivors are more likely to have a likely pathogenic variant than previous estimates of gene carrier status in MND populations; however, this may reflect breadth of our gene panel. The majority of MND patients do not undergo routine genetic testing due to variable variant penetrance, challenges with variant classification and lack of relevant treatment options for people with MND. However, recent evidence suggests that limiting genetic testing to people with presumed prior probability of having a pathogenic mutation (i.e. those with a family history and young-onset disease) may fail to capture a significant proportion of potential actionable or informative genetic mutations [45]. Our study demonstrates that slowly progressive people with MND may particularly benefit from genetic input, aiming for avoidance of prolonged and unnecessary investigation, earlier diagnosis and access to disease-specific services and care.

## Supplementary Information

Below is the link to the electronic supplementary material.

Supplementary file1 (DOCX 35 KB)

## Data Availability

Data supporting the genetic findings of this study are available within the article and supplementary material. Raw CARE-MND data are not available due to their containing information that could compromise the privacy of research participants. Further information about the CARE-MND database can be found at: https://www.caremnd.org.uk/.
