# Supplementary material for: Genotype–phenotype characterisation of long survivors with motor neuron disease in Scotland
Source: J Neurol. 2022 Dec 14;270(3):1702–12. doi: 10.1007/s00415-022-11505-0 (PMC9971124; doi:10.1007/s00415-022-11505-0)
Supplement: Supplementary file 1 — Supplementary file1 (DOCX 35 KB) [file 415_2022_11505_MOESM1_ESM.docx]

| **Gene** | **Genomic Position (GRCh37)** | **HGVS Coding**  **Transcript/Variant DNA Change** | **Amino Acid Change** | **Exon** | **Het/Hom** | **Normal Inheritance Pattern** | **Variant Type** | **dbSNP Reference** | **Number cases** | **Number controls** | **Odd Ratio Cases vs Controls**  **(p-value)** | **ACMG -AMP/ ACGS Evidence** | **Classification** | **Posterior Probability for VUS** |
| --- | --- | --- | --- | --- | --- | --- | --- | --- | --- | --- | --- | --- | --- | --- |
| ALS2 | 2:202625631CT>- | NM_020919.3:c.1085_1086delAG | p.(Glu362Alafs) | 4 | Het | AR | Frameshift |  | 1 | 0 | 91.4 (<0.01) | PVS1, PM2 | LP |  |
| ANXA11 | 10:81921766C>T | NM_001157.2:c.1105G>A | p.(Glu369Lys) | 11 | Het | AD | Missense |  | 5 | 12 | 14.3 (<0.0001) | PS4, BS1 | VUS | 0.1 |
| CCNF | 16:2499924T>C | NM_001761.2:c.1487+8T>C |  | 13 | Het | AD | Splice region |  | 1 | 0 | 91.4 (<0.01) | PS4, BP4 | VUS | 0.5 |
| CHCHD10 | 22:24110014C>T | NM_001301339.1:c.41+7G>A |  | 1 | Het | AD | Splice region |  | 3 | 9 | 10.7 (<0.001) | PS4, BS1, BP4 | VUS | 0.051 |
| DCTN1 | 2:74590116C>T | NM_004082.4:c.3529+5G>A |  | 29 | Het | AD, AR | Splice region | rs72466494 | 2 | 31 | 2.0 (0.36) | BS1, PP3 | VUS | 0.012 |
| ERBB4 | 2:212576766A>C | NM_005235.2:c.1124+9T>G |  | 9 | Het | AD | Intronic |  | 1 | 1 | 30.8 (0.02) | PS4, BP4 | VUS | 0.5 |
| FIG4 | 6:110113822A>T | NM_014845.5:c.2414A>T | p.(Asn805Ile) | 21 | Het | AD | Missense |  | 1 | 1 | 30.8 (0.02) | PS4, PM2 | LP | 0.9 |
| FIG4 | 6:110053825T>- | NM_014845.5:c.447-3delT |  | 5 | Het | AD | Splice region |  | 1 | 0 | 91.4 (<0.01) | PS4, PM2, BS1 | VUS | 0.325 |
| FUS | 16:31193960TTC>- | NM_004960.3:c.165_167delTTC |  | 3 | Het | AD | Disruptive inframe deletion |  | 1 | 1 | 30.8 (0.02) | PS4, PM4 Supporting | VUS | 0.812 |
| GRN | 17:42426926G>A | NM_002087.3:c.264+7G>A |  | 3 | Het | AD | Splice region | rs60100877 | 1 | 29 Het, 2 Hom | 1.0 (0.98) | BS1, BP4 | LB |  |
| HNRNPA2B1 | 7:26240202G>A | NM_031243.2:c.-5C>T |  |  | Het | AD | 5’ UTR |  | 1 | 0 | 91.4 (<0.01) | PS4, PM2 | LP |  |
| MAPT | 17:44067341C>T | NM_001123066.3:c.1280C>T | p.(Ser427Phe) | 8 | Het | AD | Missense |  | 2 | 7 | 8.9 (<0.01) | PS4, PP3, BS2 | VUS | 0.188 |
| NEFH | 22:29885604GAGGCCAAGTCCCCTGAGA/- | NM_021076.3:c.1975_1993delGAGGCCAAGTCCCCTGAGA |  | 4 | Het | AD, AR | Stop gained LoF |  | 7 | 162 Het, 2 Hom | 1.3 (0.49) | BS1 | VUS | 0.006 |
| NEFH | 22:29885596A>- | NM_021076.3:c.1967delA | p.(Glu656Glyfs) | 4 | Het | AD, AR | Frameshift LoF |  | 3 | 107 | 0.8 (0.76) | BS1, PM2 | VUS | 0.025 |
| NEK1 | 4:170523144C>T | NM_001199397.1:c.214+15G>A |  | 4 | Het | AD | Intronic |  | 1 | 0 | 91.4 (<0.01) | PS4, PM2, BP4 | VUS | 0.812 |
| NEK1 | 4:170398474A>C | NM_012224.2:c.2151T>G | p.(Asn717Lys) | 24 | Het | AD | Missense | rs34324114 | 1 | 0 | 91.4 (<0.01) | PS4, PP3, BS1 | VUS | 0.188 |
| NEK1 | 4:170483347C>T | NM_012224.2:c.1021G>A | p.(Ala341Thr) | 12 | Het | AD | Missense | rs189186475 | 1 | 0 | 91.4 (<0.01) | BS1, BP4 | LB |  |
| NIPA1 | 15:23086384C>- | NM_144599.4:c.28delG | p.(Ala10Argfs) | 1 | Het | AD | Frameshift LoF |  | 4 | 65 Het, 68 Hom | 0.9 (0.85) | BS1 | VUS | 0.006 |
| NIPA1 | 15:23086387CC>- | NM_144599.4:c.24_25delGG | p.(Ala9Glyfs) | 1 | Het | AD | Frameshift LoF |  | 4 | 65 Het, 68 Hom | 0.9 (0.85) | BS1 | VUS | 0.006 |
| PFN1 | 17:4849267T>A | NM_005022.3:c.351A>T | p.(Glu117Asp) | 3 | Het | AD | Missense |  | 1 | 5 | 6.1 (0.10) | BS1 | VUS | 0.006 |
| PRPH | 12:49689404G>T | NM_006262.3:c.421G>T | p.(Asp141Tyr) | 1 | Het | AD, AR | Missense | rs58599399 | 1 | 15 | 2.0 (0.50) | BS1, PP3 | VUS | 0.012 |
| SETX | 9:135152544A>- | NM_015046.5:c.6843-5delT |  | 22 | Het | AD | Splice region |  | 19 | 317 | 2.4 (<0.01) | BS1 | VUS | 0.006 |
| SETX | 9:135211747C>G | NM_015046.5:c.654G>C | p.(Lys218Asn) | 6 | Het | AD | Missense |  | 1 | 3 | 10.2 (0.05) | PS4, PM2 | LP |  |
| SETX | 9:135203176G>A | NM_015046.5:c.3809C>T | p.(Pro1270Leu) | 10 | Het | AD | Missense | rs144334281 | 1 | 1 | 30.8 (0.02) | PS4, PP3,BS1 | VUS | 0.188 |
| SETX | 9:135202468A>G | NM_015046.5:c.4517T>C | p.(Met1506Thr) | 10 | Het | AD | Missense | rs199974622 | 1 | 0 | 91.4 (<0.01) | PS4, PM2, PP3, BS3 | VUS | 0.5 |
| SOD1 | 21:33036142G>A | NM_000454.4:c.112G>A | p.(Gly38Arg) | 2 | Het | AD | Missense | rs121912431 | 1 | 0 | 91.4 (<0.01) | PS4, PM2, PP3 | LP |  |
| SOD1 | 21:33039672T>C | NM_000454.4:c.341T>C | p.(Ile114Thr) | 4 | Het | AD | Missense | rs121912441 | 4 | 0 | 293.4 (<0.001) | PS4, PM2, PP3 | LP |  |
| SPAST | 2:32366960T>- | NM_014946.3:c.1494-3delT |  | 13 | Het | AD | Splice region |  | 2 | 0 | 155.7 (<0.01) | PS4, PM2, PP3 | LP |  |
| SPG11 | 15:44887653G>A | NM_025137.3:c.4439C>T | p.(Ala1480Val) | 26 | Het | AR | Missense |  | 1 | 0 | 91.4 (<0.01) | PM2, PP3 | VUS | 0.5 |
| SPG11 | 15:44877834C>A | NM_025137.3:c.5121G>T | p.(Glu1707Asp) | 29 | Het | AR | Missense | rs145643238 | 1 | 0 | 91.4 (<0.01) | PP3 | VUS | 0.188 |
| SPG11 | 15:44856827G>A | NM_025137.3:c.7069C>T | p.(Leu2357Phe) | 39 | Het | AR | Missense | rs139334167 | 1 | 0 | 91.4 (<0.01) |  | LB |  |
| SPG11 | 15:44949428AT/- | NM_025137.3:c.733_734delAT | p.(Met245Valfs) | 4 | Het | AR | Frameshift LoF | rs312262720 | 1 | 0 | 91.4 (<0.01) |  | LP |  |
| SPG11 | 15:44952787GCTGTTACGA>- | NM_025137.3:c.276_285delTCGTAACAGC | p.(Arg93Alafs*25) | 2 | Het | AR | Frameshift LoF |  | 1 | 0 | 91.4 (<0.01) |  | LP |  |
| SQSTM1 | 5:179260112GAG>- | NM_003900.4:c.835_837delGAG | p.(Glu280del) | 6 | Het | AD | Inframe deletion |  | 1 | 0 | 91.4 (<0.01) | PS4, PM2, PM4 Supporting, PP3 | LP |  |
| SQSTM1 | 5:179263547C>T | NM_003900.4:c.835_837delGAG | p.(Glu280del) | 6 | Het | AD | Inframe deletion |  | 1 | 2 | 15.3 (0.03) | PS4, PM2, PM4 Supporting | LP |  |
| SQSTM1 | 5:179252184A>G | NM_003900.4:c.712A>G | p.(Lys238Glu) | 5 | Het | AD | Missense |  | 1 | 8 | 3.8 (0.2) | BS1 | VUS | 0.006 |
| TAF15 | 17:34171636GGCTATGGTGGAGACAGAAGTGGGGGT/- | NM_139215.2:c.1333_1359delGGCTATGGTGGAGACAGAAGTGGGGGT | p.(Ser451_Arg459del) | 15 | Het | - | Inframe deletion |  | 1 | 9 | 3.4 (0.3) | PM4, BS1 | VUS | 0.025 |
| TAF15 | 17:34171647AGACAGAAGTGGGGGTGGCTATGGTGGG/- | NM_139215.2:c.1344_1371delAGACAGAAGTGGGGGTGGCTATGGTGGG | p.(Asp449Thrfs*28) | 15 | Het | - | Frameshift LoF |  | 3 | 9 | 10.7 (<0.001) | PS4, PM2 | LP |  |
| TAF15 | 17:34171662T/- | NM_139215.2:c.1359delT | p.(Gly454Alafs) | 15 | Het | - | Frameshift LoF |  | 1 | 5 | 6.1 (0.1) | PM2, BS1 | VUS | 0.025 |
| TAF15 | 17:34171624AGAAGTGGGGGCGGCTATGGTGGAGAC/- | NM_139215.2:c.1321_1347delAGAAGTGGGGGCGGCTATGGTGGAGAC | p.(Ser451_Arg459del) | 15 | Het | - | Inframe deletion |  | 2 | 6 | 10.4 (<0.01) | PS4, PM4 | LP |  |
| TAF15 | 17:34171635CGGCTATGGTGGAGACAGAAGTGGGGGT/- | NM_139215.2:c.1332_1359delCGGCTATGGTGGAGACAGAAGTGGGGGT | p.(Gly445Alafs*28) | 15 | Het | - | Frameshift LoF |  | 2 | 6 | 10.4 (<0.01) | PS4, PM4 | LP |  |
| TAF15 | 17:34171667A>G | NM_139215.2:c.1364A>G | p.(Tyr455Cys) | 15 | Het | - | Missense |  | 1 | 0 | 91.4 (<0.01) | PS4; PM2; PP3 | LP |  |
| TAF15 | 17:34171807GGAGGAGATCGAGGAGGTTAC/- | NM_139215.2:c.1504_1524delGGAGGAGATCGAGGAGGTTAC1 | p.(Gly520_Arg526del) | 15 | Het | - | Inframe deletion |  | 1 | 8 | 3.8 (0.2) | PM4, BS1 | VUS | 0.025 |
| TAF15 | 17:34171827CGGAGGAGATCGAGGAGGTTAT/- | NM_139215.2:c.1524_1545delCGGAGGAGATCGAGGAGGTTAT | p.(Tyr508Ter) | 15 | Het | - | Stop gained LoF |  | 1 | 8 | 3.8 (0.2) | BS1 | VUS | 0.006 |
| UNC13A | 19:17799009G>A | NM_001080421.3:c.-1= |  |  | Het | AD, AR | 5’ UTR |  | 1 | 2 | 15.3 (0.03) | PS4, BP7 | VUS | 0.051 |
| VAPB | 20:56964578G>A | NM_004738.4:c.58+5G>A |  | 1 | Het | AD | Splice region |  | 1 | 0 | 91.4 (<0.01) | PS4, PM2, PP3 | LP |  |

**Supplementary Table: Variants detected in long survivors cohort**

ACMG-AMP = American College of Medical Genetics and Association for Molecular Pathology; ACGS = Association for Clinical Genomic Science; AD = autosomal dominant; AR = autosomal recessive; Het = heterozygous; Hom = homozygous; LB = likely benign; LoF = loss-of-function; LP = Likely pathogenic; UTR = untranslated region; VUS = variant of uncertain significance.

BS1 considered to be fulfilled if prevalence of variant in LBC control population is high relative to study population (OR <5).

Genotype-phenotype characterisation of long survivors with motor neuron disease, Journal of Neurology

Leighton DJ, Ansari M, Newton J, Parry D, Cleary E, Colville S, Stephenson L, Larraz J, Johnson M, Beswick E, Wong M, Gregory J, Artal JC, Davenport R, Duncan C, Morrison I, Smith C, Swingler R, Deary IJ, Porteous M, Aitman TJ, Chandran S, Gorrie GH, Pal S, the Lothian Birth Cohorts Group and the CARE-MND Consortium

Correspondence to: Dr Danielle Leighton

Address: Institute of Neurological Sciences, Queen Elizabeth University Hospital, Glasgow

E-mail: [Danielle.leighton@glasgow.ac.uk](mailto:Danielle.leighton@glasgow.ac.uk)
